# Social reactions to disclosure and perceived social support are each uniquely associated with mental health in the first 6 months following sexual assault

**DOI:** 10.3389/fpsyg.2025.1648804

**Published:** 2025-09-23

**Authors:** Siri Thoresen, Ines Blix, Grethe E. Johnsen, Tore Wentzel-Larsen, Sarah E. Ullman

**Affiliations:** ^1^Norwegian Center for Violence and Traumatic Stress Studies, Oslo, Norway; ^2^Department of Psychology, University of Oslo, Oslo, Norway; ^3^Department of Psychology, Oslo New University College, Oslo, Norway; ^4^National Centre for Emergency Primary Health Care, NORCE Research AS, Bergen, Norway; ^5^Centre for Child and Adolescent Mental Health, Eastern and Southern Norway, Oslo, Norway; ^6^Department of Criminology, Law, and Justice, University of Illinois, Chicago, Chicago, IL, United States

**Keywords:** sexual assault, PTSD, anxiety/depression, social reactions to disclosure, social support, victimization

## Abstract

**Introduction:**

It is well-known that social support is related to mental health following sexual assault (SA). Recent research suggests that social reactions to disclosure may also affect mental health; however, few studies have accounted for general social support, and most have been conducted many years after the assault. This study aimed to examine whether social reactions are uniquely linked to mental health when adjusting for perceived social support in recent victims of SA.

**Methods:**

In this cross-sectional study, participants were recruited from Sexual Assault Centers (SACs) and through social media. The sample comprised 173 female participants (mean age = 26 years, SD = 8.6) who had experienced a SA within the last 6 months and had disclosed the SA to someone. We used linear regression analyses to assess associations between social reactions to disclosure, social support, and post-traumatic stress reactions (PTSR) and anxiety/depression symptoms.

**Results:**

Although positive social reactions to disclosure were most common, negative social reactions were highly prevalent even in this early phase after SA. Negative social reactions of the Unsupportive acknowledgment type were significantly associated with more PTSR (b = 0.36, 95% CI = 0.14, 0.57), while perceived social support was significantly associated with fewer symptoms of anxiety/depression (b = −0.20, 95% CI = −0.32, −0.08).

**Conclusions:**

Social reactions and social support were differentially associated with PTSR and anxiety/depression symptoms, indicating that certain aspects of social relationships may play different roles in the recovery process. Our results call for early interventions following SA to reduce negative reactions to disclosure and facilitate the provision of positive social support to victims.

## Introduction

Sexual assault (SA) is a broad term that includes forcible, intoxicated, and attempted rape, as well as other sexual activity without consent (Tjaden and Thoennes, 2000). In the early aftermath of SA, most victims experience a high level of distress (Steenkamp et al., 2012). In contrast to many other types of traumas (Bonanno, 2005), resilience or rapid recovery does not appear to be common in SA victims. On the contrary, in women, rape is the type of trauma that carries the highest risk of post-traumatic stress disorder (PTSD) (Kessler et al., 2013). A recent meta-analysis indicated that 42% met the criteria for PTSD 1 year after the assault (Dworkin et al., 2023). The consequences of SA seem to affect mental health broadly, including an increased risk of depression and anxiety (Dworkin et al., 2017).

*Perceived (positive) social support* is closely related to mental health following traumatic events (Thoits, 2011). Social support has traditionally been conceptualized as a buffer that protects the individual from negative mental health symptom development in the face of adversity (Cohen and Wills, 1985), although longitudinal studies indicate a reciprocal relationship between PTSD symptoms and perceived support (Wang et al., 2021). Perceived support denotes the perception that emotional, cognitive, and instrumental support would be available if required (Joseph, 1999; Santini et al., 2015). Thus, through positive interaction with other people, individuals in distress may be better able to regulate emotions, achieve control over negative thought patterns, and make adaptive plans for problem solving or help seeking. Additionally, mental health symptoms may interfere with social skills, which can result in social isolation or social rejection (Kaniasty and Norris, 2008).

Most studies on mental health following traumatic events only measure the positive aspects of social relationships, such as perceived social support, even though researchers have long argued that negative aspects of social encounters also need attention. Negative and positive aspects of social interaction may be viewed as separate constructs, and negative encounters may be more harmful than positive encounters are helpful (Lincoln, 2000). Several concepts have been developed to capture such negative encounters, such as barriers to social support, social constraints, lack of support, and feeling let-down (Lepore and Ituarte, 1999; Andrews et al., 2003; Thoresen et al., 2014). One promising area of research investigates social reactions to victim disclosure of interpersonal violence, and how such reactions can affect mental health.

The concept of *social reactions* from other people post-trauma was developed within the field of interpersonal traumas such as SA and interpersonal violence, likely due to the social stigma attached to these experiences. The idea that unsupportive reactions from other people may cause harm to SA victims is not new. For example, Davis and colleagues argued that people may feel threatened or helpless when confronted with rape victims in acute distress, resulting in unsupportive behavior such as withdrawal, criticism, or inappropriate help (Davis et al., 1991). Reactions such as withdrawal or devaluation can signal a threat of exclusion, or the perception of being undesirable to others. Such social threats are likely to induce shame (Gilbert, 2000), loneliness (Thoresen et al., 2018), and withdrawal (Birkeland et al., 2020), and thereby contribute to mental health problems.

Research in this area was spurred by the development of the Social Reactions Questionnaire (Ullman, 2000), a standardized measure of social reactions to SA victims' disclosures. Social reactions comprise things people do and say in response to a victim's disclosure of SA. When considering both negative and positive social reactions to disclosure, negative reactions appear strongly linked to greater symptoms, while positive reactions seem not to provide protection, have lesser impact, or even be associated with more symptoms (Dworkin et al., 2019). Dworkin et al.'s (2019) review particularly stressed the negative impact of reactions such as controlling, distraction, and treating the survivor differently (including withdrawal). Another study, using a different measure, pointed to the negative consequences of social devaluation and discrediting (Nöthling et al., 2022). A recent meta-analysis documented a robust association between negative social reactions and psychological symptoms in SA victims (Dworkin et al., 2019). There are however two main shortcomings of the research on social reactions to disclosure. First, these studies generally do not account for social support, leaving it unclear whether social reactions represent an additional factor influencing mental health. To understand the unique potential contributions to mental health, both concepts should be assessed. To the best of our knowledge, only two previous studies have examined both, and they indicated that social support and negative reactions play different roles in the post-assault adjustment process (Ullman et al., 2007; Littleton, 2010). Second, almost all the studies on social reactions have been conducted several years after the assault, and as time passes, it may be challenging to recall specific social reactions.

Both perceived social support and social reactions are concepts related to the quality of victims' social interactions. Perceived social support typically refers to general supportive relationships, like having someone to confide in, while social reactions to disclosure focus on victimization-specific social experiences. Social reactions to disclosure may be an important additional social factor that can add to our understanding of how mental health problems evolve or are maintained following trauma in general or SA in particular.

Given the relative lack of research on aspects of social support in relationship to symptomatology soon after SA, the current study objectives were to assess the associations between social reactions to disclosure and mental health (post-traumatic stress and anxiety/depression) while adjusting for perceived social support in recent victims of SA. We hypothesized that negative social reactions would be associated with both PTSR and anxiety/depression when adjusted for perceived social support.

## Materials and methods

### Participants and procedure

This study derives data from the first wave of the TRUST study, which is an ongoing, longitudinal observational study of mental health and recovery in recent Norwegian victims of SA. The TRUST study recruited participants through Norwegian Sexual Assault Centers (SACs) and through social media, with the aim to recruit victims who had, and had not, sought help at a SAC following the recent assault. Inclusion criteria included being at least 16 years of age, understand Norwegian or English, and having experienced SA in the past 12 months. Recruitment to the study started in January 2023. The social media campaign was launched during October-November 2023 and repeated in September-October 2024.

#### Recruitment through SACs

In total, 20 of the 23 Norwegian SACs have signed a written agreement to cooperate with the TRUST study. At the SACs, victims receive medical care, forensic examinations (if suitable), and psychosocial support. SAC staff members inform patients about the ongoing study and ask for their permission to show a 2-minute information video. Alternatively, if this strategy is unsuitable in the acute treatment, the patient is given a leaflet explaining the study.

#### Recruitment through social media

The Norwegian communication agency Try designed and launched a social media recruitment campaign pro bono for this study. As most SA victims do not seek medical help in the acute phase (Thoresen and Hjemdal, 2014), the aim was to recruit individuals in the community who had experienced SA within the last year. The campaign consisted of a collaboration with social media influencers, relevant organizations, and a targeted campaign on Facebook and Instagram. The advertisement poster text was: “Have you experienced sexual assault in the past year?” By participating in the TRUST study you can help others who have similar experiences. By clicking “read more,” potential participants were directed to the study web page containing information about the study and a direct portal to the consent form.

Potential participants were asked to enter their contact information on the study web page, which was transferred encrypted to the University of Oslo Server for sensitive data. Those who entered their contact information received a text message or an e-mail (depending on their preference) 1–3 days later with a link to a web site presenting the study information (https://trust.nkvts.no/eng/). Individuals willing to participate were asked to consent through a secure identity confirmation system (“BankID”). Participants could choose between web questionnaires or telephone interviews. Interviews were conducted by members of the TRUST research team (healthcare personnel with experience in research interviews and one master of science in psychology). Upon completion of the questionnaire/interview, data was transferred encrypted (without local storage) to the University of Oslo Server for sensitive data (TSD).

A temporary data file was created in May 2024, at which time we had achieved a sufficient sample size to answer our research questions (*N* = 519). We selected participants who had experienced SA during the last 6 months (*n* = 201). Unfortunately, we had to exclude the few participants of male sex due to the very low number (*n* = 4, none of whom identified as female). Of the remaining 197 participants, the 173 reporting that they had disclosed the SA to someone (in addition to SAC personnel) constituted our final sample.

### Ethical considerations

The TRUST study recruits individuals recently exposed to SA and asks them to share highly sensitive information. Although trauma research participants most often value the experience positively (Jaffe et al., 2015), the study team has taken steps to counteract any potential negative experiences: 1. Potential participants could use the time they needed to consider participation after being presented with the study information; 2. SAC staff and other health personnel were not informed about participation, and participation in the study could not affect health services in any way; 3. Potential adverse reactions to the study were assessed, and those who needed support were offered consultation(s) at an independent stress and trauma clinic; 4. Participants could choose if they wanted to complete an online questionnaire, or be interviewed by one of the researchers on telephone. Each participant has signed an informed consent, and the TRUST study is approved by the Regional Committee for Medical and Health Research (REK 398925/2022) in accordance with Norwegian law. The data file was deidentified and data storage is approved by SIKT (Norwegian Agency for Shared Services in Education and Research).

#### Lived experience collaboration

The TRUST study was designed in close collaboration with individuals with lived experience (survivors) and people working with survivors, including representatives of the Norwegian Association against Sexual Abuse and the Norwegian PTSD Association, and professionals working with SA victims (such as at sexual assault resource and self-help centers and at SACs).

### Measures

*Social reactions to disclosure*: Participants were asked if they had disclosed the SA to anyone (in addition to SAC personnel). The 173 participants who had disclosed to at least one person were administered the Social Reactions Questionnaire—shortened (SRQ-S) (Ullman et al., 2017). This instrument assesses frequency of reactions to disclosure from the social network or from formal providers received within three subdomains; turning against (blame, stigma and infantilizing); unsupportive acknowledgment (control, distract, egocentric); and positive reactions (emotional support, tangible aid). The shortened version has good construct validity and internal consistency at baseline and over time. The instrument was translated in collaboration with the developer Sarah Ullman and a Norwegian translation agency. Two adaptations were made due to cultural or translational difficulties: The question “Reassured you that you are a good person” (SRQ-S no 2) was replaced with “Told you it was not your fault” (SRQ no 1) and the question “Made decisions or did things for you” (SRQ-S no 8) was not included. The Norwegian version of the SRQ-S comprised 15 items (see Table 1) scored on a scale from “Never” (0) to “Always” (4). In our sample, Cronbach's alphas were 0.86 for the Turning against subscale, 0.72 for Unsupportive acknowledgment, and 0.74 for Positive reactions.

**Table 1:** Social reactions to disclosure in recent female SA victims (*n* = 171–173).

**Social reactions to disclosure**	**Sometimes/often/very often % (*n*)**
**Turning against subscale**	
Told you that you were irresponsible/not cautious enough	31.8 (55)
Told you that you could have done more to prevent this experience from occurring	29.5 (51)
Treated you differently in some way than before you told them that made you feel uncomfortable	34.1 (59)
Avoided talking to you or spending time with you	20.8 (36)
Treated you as if you were a child or somehow incompetent	19.2 (33)
Made you feel like you didn't know how to take care of yourself'	28.5 (49)
**Unsupportive acknowledgment subscale**	
Tried to take control of what you did/decisions you made	47.7 (82)
Told you to go on with your life	40.4 (69)
Told you to stop thinking about	35.4 (61)
Has been so upset that they needed reassurance from you	39.0 (67)
Expressed so much anger at the perpetrator that you had to calm them down	40.5 (70)
**Positive reactions subscale**	
Reassured you that it wasn't your fault	85.0 (147)
Comforted you by telling you it would be all right or by holding you	72.7 (125)
Provided information and discussed options	65.3 (113)
Helped you get information of any kind about coping with the experience	52.0 (90)

*Current perceived social support* was measured by Duke-UNC Functional Social Support Questionnaire (FSSQ) (Broadhead et al., 1988). The scale comprises seven items: Having people around you that care about what happens to you; receiving love and care from close ones; having someone to talk to about work/school/study problems; having trusted people you can talk to about personal or family problems; being included in social activities with others; getting useful advice about important things in life; and being cared for when sick. Respondents are asked to evaluate each item on a 5-point scale: “As much as I would like” (5); “Almost as much as I would like” (4); “Somewhat, but would like more” (3); “Less than I would like” (2); and “Much less than I would like” (1). The FSSQ has been shown to be valid and reliable (Broadhead et al., 1988). Cronbach's alpha in the present study was 0.91.

*PTSR since the assault* were measured by the International Trauma Questionnaire (ITQ), (Cloitre et al., 2018), Norwegian version by Bækkelund et al. (2019). The ITQ corresponds to ICD-11 diagnosis of PTSD, and comprises two items for each subscale (re-experiencing, avoidance, and hypervigilance), measured on a scale from “Not at all” (0) to “Extremely” (4). Participants were asked to report PTSR symptoms related to the index SA (the assault that preceded their attendance to a SAC/their participation in the study). We used the recommended scoring method to indicate a clinical level of symptoms: A score of ≥2 (“moderately”) on at least one item from each of the three subscales. Cronbach's alpha in the present study was 0.80.

*Anxiety and Depression symptoms* (last week) were measured by a 10-item version of the Hopkins Symptom Checklist-25 (HSCL) (Derogatis et al., 1974), measuring symptoms of depression and anxiety (suddenly scared for no reason; feeling fearful; faintness, dizziness, or weakness; feeling tense or uneasy; blaming yourself for things; feeling blue; feelings of worthlessness; feeling everything is an effort; feeling hopeless about the future; problem sleeping) on a scale from “Not bothered” (1) to “Bothered a great deal” (4). This abbreviated version of HSCL has shown good psychometric properties and has previously been found to correlate highly (*r* = 0.97) with the HSCL-25 in a general population sample. We used their recommended cutoff value of >1.85 to indicate a clinically significant level of symptoms (Tambs and Moum, 1993). For this study, Cronbach's α was 0.89.

Control variables included victimization history, assault characteristics, help-seeking, and socio-demographic variables. *Victimization history* was measured by five questions: *Before age 18*: (1) Did a parent or caregiver ever slap you repeatedly, beat you, or otherwise attack or harm you?; (2) Did someone do sexual things with you, or made you do sexual things with them, when you could not say no, were forced or pressured?; *From age 18*: (3) Have you previously experienced one or more SA(s)?; (4) Have you ever been kicked, hit, beat up or otherwise physically harmed by a romantic partner/spouse?; (5) Have you ever been kicked, hit, beat up or otherwise physically harmed by someone else in your family, an acquaintance or someone else? Response format for all items were “yes” or “no.” Questions were adapted from the Stressful Life Event Screening Questionnaire (SLESQ) (Goodman et al., 1998), Norwegian version (Thoresen and Øverlien, 2009) and The Child and Adolescent Trauma Screen 2 (CATS-2) (Sachser et al., 2022); Norwegian version by Norwegian Center for Violence and Traumatic Stress Studies (https://www.nkvts.no//content/uploads/2022/11/TRAUME-OG-PTSD-SCREENING-TRAPS-II-norsk.pdf). Item 3 was created for this study. Following the results from a Swedish study of recent rape victims (Tiihonen Möller et al., 2014), we constructed a dichotomous variable indicating 0–1 vs. 2 or more “yes” answers to categories of previous victimization experiences.

*Assault characteristics* included: (1) Relationship to the perpetrator (dichotomous dummy variable indicating “close” (current/ex romantic partner or family member) vs. “other” (unknown, known less than 24 h, friend/colleague/fellow student, authority person or other); (2) completed penetration (yes or no); (3) the perpetrator's use of physical force or threats to harm (e.g., holding down or threats of violence) (yes or no); (4) the perpetrator's actual use of physical violence during the assault (yes or no); and (5) victim intoxication at the time of the assault (being so sleepy, unconscious, or drunk that the individual could not consent or oppose to what was happening) (yes or no). Time since assault was recorded as a categorical variable: 1–6 days, 1–2 weeks, 3–4 weeks, 1–3 months, 3–6 months.

*Help-seeking at a SAC*

Participants were asked if they had sought help at a SAC as a result of the recent SA (yes/no).

*Sociodemographic variables* included age, biological sex (“What is your biological sex”) and gender (“Do you identify as another gender?” and “If yes, which gender?”), national background (born in Norway by Norwegian-born parents or not). Among the 173 participants of female sex, all self-identified as women. Education level indicated whether or not the participant had completed 13 years of education. A dichotomous variable of self-perceived financial situation was constructed indicating whether or not the participants perceived their financial situation to be worse than most people.

### Statistical analyses

We used Pearson's *r* to examine correlations between continuous variables and independent sample *t*-tests to assess differences between SAC seekers and non-SAC-seekers on mental health, social reactions, and social support. To assess associations between the three SRQ subscales, social support and mental health, we performed a series of linear regression analyses, separately for post-traumatic stress and anxiety/depression. First, unadjusted associations are presented. Secondly, all variables were included in an adjusted model (SRQ subscales, social support, age, national background, help-seeking, time since assault, assault characteristics, and victimization history). In the adjusted model, multicollinearity was assessed by variance inflation factor (VIF), with the most commonly used thresholds of 5 and 10 used for indications of problematic and highly problematic multicollinearity (Kim, 2019). Sensitivity analyses were conducted to determine if adjustment for education level and perceived financial situation would substantially alter the regression coefficients of the social reactions and social support variables. Regressions were performed without bootstrapping, but due to observed skewness in two of the SRQ subscales, we performed bootstrapping (10,000 bootstrap replications) and compared confidence intervals with BC_a_ (bias corrected and accelerated) and without bootstrapping.

“Half rule” was used for scale variables, i.e., mean scores were calculated based on the means of valid items within each scale (Fairclough, 2010) as long as at least half the questions were answered. No participant had more than three missing items on scale variables, and mean scores were calculated for all participants. Because of few missing values, 172 of the 173 participants were included in the regression analyses. All tests were two-tailed, with a significance level of *p* < 0.05. Statistical analyses were performed using IBM SPSS statistics for Windows, version 29, bootstrapping was performed in the R package boot.

This study was preregistered in OSF: https://osf.io/nx437/. We made no major changes to the planned analyses, except that we performed bootstrapping due to observed skewness in two of the SRQ subscales and included the demographic variables age and national background in the SRQ subscales regressions.

## Results

The 173 female participants had a mean age of 26 years (SD = 8.6, range 16–71), 86.7% (*n* = 150) was born in Norway by Norwegian-born parents, 85.0% (*n* = 147) had completed 13 years of education, and 29.1% (*n* = 48) reported that they had a financial situation “worse than most people.” The perpetrator was a partner, ex-partner or family member in 17.4% (*n* = 30) of the SAs. Most assaults included sexual penetration (80.9%, *n* = 140). The perpetrator used physical force or threats to harm in 38.7% (*n* = 67), and actual physical violence in 25.4% (*n* = 44) of the assaults. About half (50.9%, *n* = 88) of the participants reported that they were not able to consent or defend themselves due to intoxication. In total, 47.4% (*n* = 82) had two or more experiences of previous physical violence and/or SA. In total, 53.8% (*n* = 93) had sought help at a SAC after the assault. Most participants (88.4%, *n* = 153) responded on a web questionnaire, while the rest (11.6%, *n* = 20) chose to be interviewed by telephone.

Symptom levels were very high among participants. The mean PTSR score was 2.4 (SD = 0.9) on a scale from 0 to 4, where a score of 2 indicates “moderately” and 3 indicates “quite a bit.” The mean anxiety/depression score was 3.0 (SD = 0.7) on a scale from 1 to 4, where 3 indicates “bothered quite a bit.” Symptom levels showed little variation according to number of months since the SA for both PTSR and anxiety/depression (mean PTSR scores ranged from 2.4 to 2.5, and mean anxiety/depression scores ranged from 2.9 to 3.1, depending on the number of months since SA). A clinically significant level of PTSR was observed in 68.6% of participants (*n* = 118), and in 69.2% (*n* = 74) of those for whom more than a month had passed since the SA. A clinically significant level of anxiety/depression symptoms was observed in 90.8% (*n* = 157) of participants. Symptom levels were significantly higher in participants who had sought help at a SAC (mean PTSR = 2.6, SD = 0.8) and mean anxiety/depression = 3.1, SD = 0.6) compared to those who had not (mean PTSR = 2.3, SD = 1.0) and mean anxiety/depression = 2.8, SD = 0.8), *t*-test *p* < 0.001 for both comparisons. Neither the negative social reactions nor social support differed significantly between SAC- and non-SAC seekers (*t*-test *p*-values = 0.313–0.758). However, SAC-seekers reported more positive social reactions (SAC-seekers mean = 2.4, SD = 0.9; non-SAC-seekers mean = 1.9, SD = 0.9, *t*-test *p*-value < 0.001).

### Social reactions to disclosure

Our sample constituted only participants who had disclosed the SA to someone. Participants reported a variety of social reactions to disclosure of the SA (Table 1). Positive reactions were more frequently reported than negative reactions. Particularly, participants perceived that other people had reassured them that what happened wasn't their fault. The most frequently reported negative social reactions were in the “Unsupportive acknowledgment subscale,” namely others trying to take control, expressing excessive anger, and telling the victim to go on with her life. One of five to one of three victims reported negative social reactions of the “Turning against” type. For example, about a third of the victims reported that other people had treated them differently in a negative way after disclosure.

Correlations between scale variables are displayed in Table 2. The two SRQ negative reactions subscales were strongly correlated, and both were moderately and negatively associated with social support. The SRQ subscale “Turning against” was negatively associated with the SRQ positive reactions subscale, while the SRQ subscale “Unsupportive acknowledgement” was not significantly associated with positive reactions. PTSR were moderately positively associated with anxiety/depression symptoms. The *p*-values with adjustment for multiple tests within this group of 15 correlation coefficients were computed using the Holm procedure. Then, the *p*-value for scale variable 1 vs. 6 was 0.003, 2 vs. 3 was 0.012, 2 vs. 4 0.028, 4 vs. 6 was 0.005. All other significant correlations still had *p* < 0.001.

**Table 2:** Correlations between scale variables (Pearson's *r*), range, means (M) and standard deviations (SD).

**Scale variables**	**2**		**3**		**4**		**5**		**6**	
	***r***	***p*** **-value**	***r***	***p*** **-value**	***r***	***p*** **-value**	***r***	***p*** **-value**	***r***	***p*** **-value**
1 Posttraumatic stress reactions, scale range: 0–4, M = 2.4, SD = 0.9	0.61	< 0.001	0.30	< 0.001	0.39	< 0.001	−0.03	0.738	−0.27	< 0.001
2 Anxiety/depression, scale range: 1–4, M = 3.0, SD = 0.7			0.23	0.002	0.20	0.007	0.07	0.345	−0.29	< 0.001
3 SRQ subscale 1, scale range: 0–4, M = 0.9, SD = 0.8					0.70	< 0.001	−0.30	< 0.001	−0.41	< 0.001
4 SRQ subscale 2, scale range: 0–4, M = 1.2, SD = 0.9							−0.03	0.716	−0.25	< 0.001
5 SRQ subscale 3, scale range: 0–4, M = 2.1, SD = 0.9									0.36	< 0.001
6 Social support: scale range: 1–5, M = 3.6, SD = 1.0										

*N* = 173 female SA victims.

### Associations between social reactions, social support, and PTSR

Univariably, both SRQ negative reactions subscales, as well as social support, were significantly associated with PTSR, while SRQ positive reactions subscale was not (Table 3). In the adjusted model, only the SRQ negative reactions subscale “Unsupportive acknowledgment” remained significantly associated with PTSR. The association between social support and PTSR was not statistically significant in the adjusted model. No indications of problems with multicollinearity were detected, VIFs were 2.63,2.23, 1.51 and 1.42 for the SRQ variables and social support, respectively.

**Table 3:** Unadjusted and adjusted associations between independent variables and post-traumatic stress symptoms using linear regressions.

**Independent variables**	**Unadjusted**		**Adjusted** ^**a**^	
	**Regression coefficient and 95% CI**	***P*** **-value**	**Regression coefficient and 95% CI**	***P*** **-value**
SRQ turning against	0.32 (0.17, 0.48)	< 0.001	−0.05 (−0.28, 0.19)	0.705
SRQ unsupportive acknowledgment	0.41 (0.26, 0.56)	< 0.001	0.36 (0.14, 0.57)	0.001
SRQ positive reactions	−0.02 (−0.17, 0.12)	0.738	0.03 (−0.13, 0.19)	0.733
Social support	−0.24 (−0.37, −0.11)	< 0.001	−0.13 (−0.28, 0.01)	0.068

^a^Additionally adjusted for age, national background, help-seeking (at a SAC), time since assault, assault characteristics (assaulted by someone close, penetration, force/threats, physical violence and victim intoxication) and victimization history.

*N* = 172 female SA victims, *R*^2^ for adjusted model = 0.29 (adjusted *R*^2^ = 0.21).

### Associations between social reactions, social support, and anxiety/depression

As with PTSR, both SRQ negative reactions subscales, as well as social support, were significantly associated with anxiety/depression symptoms in the unadjusted models (Table 4). However, only social support remained significantly related to less anxiety/depression in the adjusted model. The SRQ “Positive reactions” subscale was significantly associated with more anxiety/depression symptoms in the adjusted model. The VIFs were the same as for PTSR.

**Table 4:** Unadjusted and adjusted associations between independent variables and anxiety/depression symptoms using linear regressions.

**Independent variables**	**Unadjusted**		**Adjusted** ^**a**^	
	**Regression coefficient and 95% CI**	***P*** **-value**	**Regression coefficient and 95% CI**	***P*** **-value**
SRQ turning against	0.20 (0.07, 0.32)	0.002	0.10 (−0.09, 0.29)	0.303
SRQ unsupportive acknowledgment	0.17 (0.05, 0.30)	0.007	0.05 (−0.12, 0.23)	0.564
SRQ positive reactions	0.06 (−0.06, 0.17)	0.345	0.16 (0.03, 0.29)	0.019
Social support	−0.21 (−0.31, −0.11)	< 0.001	−0.20 (−0.32, −0.08)	< 0.001

^a^Additionally adjusted for age, national background, help-seeking (at a SAC), time since assault, assault characteristics (assaulted by someone close, penetration, force/threats, physical violence and victim intoxication) and victimization history. *N* = 172 female SA victims. *R*^2^ for adjusted model = 0.23 (adjusted *R*^2^ = 0.14).

Appendices 1, 2 display results for all variables included in the adjusted model for Tables 3, 4.

Sensitivity analyses were performed to examine whether adjustment for education level and perceived financial situation would result in any noticeable change in the regression coefficients for SRQ and social support with PTSR and anxiety/depression, respectively. No large changes were observed (not displayed) and the same factors remained significantly associated with the outcome variables. No major differences were identified when regression analyses were compared with bootstrap results. In some cases, confidence intervals were somewhat wider with bootstrap (see electronic Appendix 3).

## Discussion

The main results of this study showed that social reactions to disclosure were associated with PTSR in recent victims of SA, even when adjusted for perceived social support and that social support was significantly associated with less anxiety/depression symptoms. Our findings suggest that other people's reactions to victims' disclosure are closely associated with mental health difficulties, particularly PTSR, even in the early aftermath of the assault.

Consistent with previous research, positive social reactions were most common (Filipas and Ullman, 2001; Jónsdóttir et al., 2024). However, many victims received reactions that are considered negative, such as other people trying to take control, telling the victim to stop thinking about it, or treating the victim differently in a negative way. Such negative reactions are thought to convey to the victim; for example, that she is somehow responsible for the assault, is unable to take care of herself, is over-reacting, or is damaged by the assault (Dworkin et al., 2019). In line with previous research (Relyea and Ullman, 2015) *unsupportive acknowledgment* were more common than *turning against* reactions and were also more strongly associated with post-traumatic stress reactions (PTSR). These types of responses may be unhelpful not because they involve denial or victim-blaming, but because they lack sufficient sensitivity to the victim's needs—such as when others attempt to take control of the situation or urge the victim to stop thinking about the experience.

Based on previous literature, we hypothesized that negative social reactions would be associated with both PTSR and anxiety/depression beyond the effects of previous victimization, assault characteristics, and time since assault, and that the social reactions-mental health links would remain statistically significant, even when adjusting for perceived social support. Our findings partly supported these hypotheses. As expected, negative social reactions to disclosure, particularly Unsupportive acknowledgment, were significantly associated with higher levels of PTSR in this early phase after SA, even when adjusted for social support. Thus, efforts to reduce these negative social reactions, even relatively soon after SA, appear crucial for achieving symptom relief—particularly taking into account the high symptom levels reported both here and in previous studies (Steenkamp et al., 2012).

However, social support, but not negative social reactions, was significantly associated with anxiety/depression in the adjusted model. This finding was unexpected, as a recent meta-analysis showed that most studies report associations between social reactions and a broad range of psychopathology, including anxiety and depression (Dworkin et al., 2019). However, there are exceptions. For example, one of the few studies included in the meta-analysis that focused on recent SAs, found that social reactions were associated with fear, but not with PTSD symptoms nor depression (Orchowski and Gidycz, 2015). Further, an intervention study aimed at improving social reactions, indicated reduced symptoms of PTSD, but not of depression, in subsequent SA victims (Edwards et al., 2021). Moreover, in a study on reasons for non-disclosure of SA, specific reasons for non-disclosure were related to PTSD, but not to depression (Carson et al., 2020). These findings suggest that social reactions and social support may have different associations with different symptoms. Further research is needed to understand how social reactions affect different types of symptoms over time, and across geographic locations and samples.

Our finding of evidence that social support, but not social reactions, was associated with anxiety/depression in the adjusted model needs further consideration. It is often assumed that negative aspects of social interactions, such as feeling let-down, social support barriers, interpersonal friction, and social constraints, have a greater detrimental impact on mental health than the (potentially) protective effects of social support (Zoellner et al., 1999; Andrews et al., 2003; Thoresen et al., 2014). In our study, the regression coefficient for social support was largely unaffected by adjustment for social reactions, indicating that positive aspects of social interactions, more than negative aspects, were important for anxiety/depression. A few previous studies have simultaneously investigated social support and social reactions to disclosure of SA; and they indicate that social support and negative disclosure reactions may play unique roles in the post-assault recovery process (Ullman et al., 2007; Littleton, 2010). As Littleton suggests, social support may be key to preserving a sense of self-worth following trauma, which may be specifically important for depression symptoms (Littleton, 2010). Also, in the case of other types of trauma, positive and negative aspects of social interactions may have unique impacts on mental health (Charuvastra and Cloitre, 2008; Thoresen et al., 2014), representing separate pathways to symptoms or recovery. Future research should aim to identify specific aspects of social interactions, both positive and negative, that can inform treatment and prevention of symptomatology for victims of SA.

This study was cross-sectional and therefore could not determine the temporal relationships between social support, social reactions, and mental health outcomes. While a lack of social support and negative social reactions may contribute to poorer mental health, it is also possible that individuals with more severe symptoms receive less support and more negative reactions—or perceive support and reactions more negatively. The potential temporal relationship between negative social reactions to disclosure and mental health is unclear due to the lack of longitudinal studies, expect for one study indicating a bidirectional relationship (Ullman and Peter-Hagene, 2016). Concerning social support, a recent meta-analysis similarly suggests a reciprocal relationship between social support and mental health (Wang et al., 2021). Some studies indicate that social support may have a buffering effect in the early stages following trauma, while mental health problems may contribute to a decline in social support over time (Kaniasty and Norris, 2008). An alternative explanation is that the commonly observed associations between social factors and mental health may not reflect causality but instead be driven by unrecognized third variables.

In sum, results underscore the potential importance of the social environment following SA. This conclusion is consistent with many previous studies pointing to the importance of post-trauma factors for recovery (Brewin et al., 2000; Trickey et al., 2012; Maercker and Hecker, 2016) and underscores the need for social-ecological models to understand and change social and systemic conditions for recovery (Campbell et al., 2009; Jewkes et al., 2022).

User panel/lived experience: The results and interpretations in this study were discussed with the TRUST study's lived experience representatives. They related the study findings on social reactions and social support to their personal and professional experiences and gave examples of how negative social reactions can be truly hurtful. For future research, it was suggested to further our understanding of how negative social reactions may influence shame. Additionally, they addressed the need to understand the role of intimate partner violence in the social context of recovery after SA.

### Limitations

The current study relied on cross-sectional data, and causal relationships between social reactions, social support, and psychological symptoms could not be determined. The small number of male participants prevented us from examining male victims and potential gender differences. Selection bias is likely, and the sample cannot be considered representative of all SA victims. Specifically, it is likely that victims who have disclosed the assault are overrepresented in our study. All measures were based on retrospective self-report, with the risk of biases or forgetting. The high level of psychological symptoms in our sample may have affected the participants' responses, posing a potential threat to the validity of the data.

Unmeasured variables may have affected our results, for example we lacked information about pre-assault mental health problems. It should also be noted that social reactions characterized by researchers as “negative” or “positive” are not always perceived as such by victims (Dworkin et al., 2018). Although the correlations between social reactions subscales and social support were only moderate, these constructs are likely not entirely independent and may potentially influence one another in complex ways that were beyond the scope of the present study. For instance, negative social reactions could shape an individual's perception of available social support and vice versa. Lastly, the associations between social variables and mental health outcomes were observed within a certain time period after the SA (< 6 months), and such associations may potentially change over time. Despite these limitations, this study had several strengths included recruitment shortly after SA (within 6 months), with both help-seeking and non-help-seeking participants, the ability to investigate social reactions and social support simultaneously, and a sufficient sample size with very little missing data.

### Conclusions

Our results show that social factors may be of high importance for the recovery process in the early aftermath of SA. Negative social reactions to disclosure, particularly unsupportive acknowledgment, were associated with more post-traumatic stress reactions while general social support was associated with less anxiety/depression symptoms.

Early interventions for SA victims could, where appropriate, include consultations with family or friends, to achieve more helpful reactions from the social network. Specifically, family and friends may be encouraged to refrain from certain negative reactions, such as taking control, overreacting, or urging the SA victim to stop thinking about what happened, while fostering general social support by being present and engaging in shared activities. Clinicians can assist SA victims in finding safe ways to disclose, prepare for, and deal with potential unhelpful social reactions. A previous study has shown preliminary support for social network interventions in SA victims (Edwards et al., 2022), but more research is needed in this area. Such interventions can be implemented in SACs or in other sexual assault support centers as well as in college/university clinics. Additionally, professionals across disciplines, such as law enforcement, the justice system, victim services, and healthcare, should be informed about supportive and potentially harmful responses to victims' disclosures of sexual assault.

Research is needed to understand how SA interferes with social relations, and to pinpoint the specific aspects of social relationships that are of importance for specific mental health symptoms at different time periods after SA. The mechanisms involved in the relationship between social reactions and mental health, such as for example shame and self-blame, also need further exploration. Finally, there is also a need to develop and test interventions focusing on navigating and improving social relationships in the aftermath of SA.

## Data Availability

The datasets presented in this article are not readily available because the data set includes highly sensitive information and there are legal restrictions to sharing data. Researchers can get access to the data set, but only after applying to (and acceptance from) the Regional Committee for Medical and Health Research. Requests to access the datasets should be directed to siri.thoresen@nkvts.no.
