# Supplementary material for: Social reactions to disclosure and perceived social support are each uniquely associated with mental health in the first 6 months following sexual assault
Source: Front Psychol. 2025 Sep 23;16:1648804. doi: 10.3389/fpsyg.2025.1648804 (PMC12500567; doi:10.3389/fpsyg.2025.1648804)
Supplement: Supplementary file 1 [file Table_1.docx]

Appendix 1 Table 3 displaying all variables:
Unadjusted and Adjusted Associations between Independent Variables and Posttraumatic Stress symptoms using Linear Regressions.

|  | Unadjusted | | Adjusted | |  |  |
| --- | --- | --- | --- | --- | --- | --- |
| Independent variables | Regression coefficient and 95% CI | P value | Regression coefficient and 95% CI | P value | VIF^a^ |  |
| Age | 0.02  (0.01, 0.04) | 0.006 | 0.01  (-0.002, 0.03) | 0.077 | 1.31 |  |
| National background | 0.08  (-0.33, 0.49) | 0.700 | 0.02  (-0.35, 0.39) | 0.899 | 1.07 |  |
| Help-seeking (yes vs no) | 0.36  (0.08, 0.63) | 0.010 | 0.35  (-0.07, -0.62) | 0.015 | 1.28 |  |
| Time since assault: Reference 3-6 months |  | 0.970 |  | 0.615 |  |  |
| 1-6 days | -0.13  (-0.58, 0.32) | 0.569 | -0.05  (-0.49, 0.40) | 0.833 | 1.58 |  |
| 1-2 weeks | -0.13  (-0.59, 0.34) | 0.593 | -0.23  (-0.67, 0.21) | 0.306 | 1.46 |  |
| 3-4 weeks | -0.12  (-0.61, 0.37) | 0.636 | -0.13  (-0.58, 0.32) | 0.575 | 1.33 |  |
| 1-3 months | -0.07  (-0.42, 0.29) | 0.706 | 0.09  (-0.23, 0.42) | 0.568 | 1.55 |  |
| Assaulted by someone close | 0.06  (-0.30, 0.43) | 0.736 | -0.16  (-0.53, 0.20) | 0.379 | 1.30 |  |
| Penetration | 0.06  (-0.29, 0.41) | 0.730 | -0.03  (-0.37, 0.31) | 0.849 | 1.17 |  |
| Physical force/ threats to harm | 0.32  (0.04, 0.60) | 0.024 | 0.05  (-0.26, 0.35) | 0.767 | 1.46 |  |
| Physical violence | 0.48  (0.17, 0.79) | 0.003 | 0.19  (-0.15, 0.53) | 0.280 | 1.51 |  |
| Victim intoxication | -0.24  (-0.52, 0.03) | 0.081 | -0.04  (-0.32, 0.24) | 0.761 | 1.30 |  |
| Victimization history | 0.51  (0.24, 0.78) | <0.001 | 0.28  (-0.01, 0.57) | 0.059 | 1.39 |  |
|  |  |  |  |  |  |  |
| SRQ Turning against | 0.32  (0.17, 0.48) | <0.001 | -0.05  (-0.28, 0.19) | 0.705 | 2.63 |  |
| SRQ Unsupportive acknowledgement | 0.41  (0.26, 0.56) | <0.001 | 0.36  (0.14, 0.57) | 0.001 | 2.23 |  |
| SRQ Positive reactions | -0.02  (-0.17, 0.12) | 0.738 | 0.03  (-0.13, 0.19) | 0.733 | 1.51 |  |
| Social support | -0.24  (-0.37, -0.11) | <0.001 | -0.13  (-0.28, 0.01) | 0.068 | 1.42 |  |

N = 172 female SA victims
R^2^ for adjusted model = 0.29 (adjusted R^2^ = 0.21)
^a^VIF = Variance Inflation Factor
