# Supplementary material for: Social reactions to disclosure and perceived social support are each uniquely associated with mental health in the first 6 months following sexual assault
Source: Front Psychol. 2025 Sep 23;16:1648804. doi: 10.3389/fpsyg.2025.1648804 (PMC12500567; doi:10.3389/fpsyg.2025.1648804)
Supplement: Supplementary file 2 [file Table_2.docx]

Appendix 2 Table 4 displaying all variables:
Unadjusted and Adjusted Associations between Independent Variables and Anxiety/depression using Linear Regressions.

|  | Unadjusted | | Adjusted | |  |
| --- | --- | --- | --- | --- | --- |
| Independent  variables | Regression coefficient and 95% CI | P value | Regression coefficient and 95% CI | P value | VIF^a^ |
| Age | 0.01  (0.001, 0.03) | 0.035 | 0.01 (-0.003, 0.02) | 0.112 | 1.31 |
| National background | 0.11 (-0.21, 0.43) | 0.494 | 0.05 (-0.26, 0.35) | 0.767 | 1.07 |
| Help-seeking (yes vs no7) | 0.21  (-0.01, 0.42) | 0.062 | 0.11 (-0.12, 0.33) | 0.362 | 1.28 |
| Time since assault: Reference 3-6 months |  | 0.370 |  | 0.329 |  |
| 1-6 days | 0.31  (-0.04, 0.66) | 0.086 | 0.38  (0.01, 0.75) | 0.042 | 1.58 |
| 1-2 weeks | 0.11  (-0. 25, 0.48) | 0.542 | 0.05  (-0.31, 0.41) | 0.788 | 1.46 |
| 3-4 weeks | -0.03  (-0.41, 0.36) | 0.893 | 0.05  (-0.32, 0.42) | 0.803 | 1.33 |
| 1-3 months | -0.03  (-0.31, 0.25) | 0.835 | 0.04  (-0.23, 0.31) | 0.747 | 1.56 |
| Assaulted by someone close | 0.04  (-0.25, 0.33) | 0.783 | 0.01  (-0.29, 0.31) | 0.940 | 1.30 |
| Penetration | -0.07  (-0.35, 0.21) | 0.608 | -0.10  (-0.38, 0.18) | 0.476 | 1.17 |
| Physical force/threats to harm | 0.10  (-0.12, 0.32) | 0.380 | -0.04  (-0.29, 0.21) | 0.726 | 1.46 |
| Physical violence | 0.08  (-0.17, 0.33) | 0.528 | -0.06  (-0.34, 0.22) | 0.678 | 1.51 |
| Victim intoxication | -0.13  (-0.35, 0.08) | 0.224 | -0.10  (-0.33, 0.13) | 0.409 | 1.30 |
| Victimization history | 0.34  (0.13, 0.56) | 0.002 | 0.14  (-0.10, 0.38) | 0.253 | 1.39 |
| SRQ Turning against | 0.20  (0.07, 0.32) | 0.002 | 0.10  (-0.09, 0.29) | 0.303 | 2.63 |
| SRQ Unsupportive acknowledgement | 0.17  (0.05, 0.30) | 0.007 | 0.05  (-0.12, 0.23) | 0.564 | 2.23 |
| SRQ Positive reactions | 0.06  (-0.06, 0.17) | 0.345 | 0.16  (0.03, 0.29) | 0.019 | 1.51 |
| Social support | -0.21  (-0.31, -0.11) | <0.001 | -0.20  (-0.32, -0.08) | <0.001 | 1.42 |

N = 172 female SA victims
R^2^ for adjusted = 0.23 (adjusted R^2^ = 0.14)
^a a^VIF = Variance Inflation Factor
