# Supplementary material for: Social reactions to disclosure and perceived social support are each uniquely associated with mental health in the first 6 months following sexual assault
Source: Front Psychol. 2025 Sep 23;16:1648804. doi: 10.3389/fpsyg.2025.1648804 (PMC12500567; doi:10.3389/fpsyg.2025.1648804)
Supplement: Supplementary file 3 [file Table_3.docx]

Appendix 3 95% Confidence Intervals (CI) With and Without Bootstrap for the Unadjusted and Fully Adjusted Linear Regression Analyses

| Independent variable | Unadjusted | | | | Adjusted | | | |
| --- | --- | --- | --- | --- | --- | --- | --- | --- |
|  | Lower | | Upper | | Lower | | Upper | |
|  | Without bootstrap | With bootstrap | Without bootstrap | With bootstrap | Without bootstrap | With bootstrap | Without bootstrap | With bootstrap |
| *SRQ Turning Against* |  |  |  |  |  |  |  |  |
| Assaulted by someone close^a^ | 0.03 | 0.03 | 0.70 | 0.78 | -0.07 | -0.09 | 0.59 | 0.67 |
| Unwanted penetration | -0.69 | -0.72 | -0.05 | -0.07 | -0.73 | -0.73 | -0.09 | -0.10 |
| Physical force/threats to harm | -0.05 | -0.05 | 0.47 | 0.49 | -0.22 | -0.22 | 0.36 | 0.37 |
| Physical violence | -0.02 | -0.05 | 0.56 | 0.64 | -0.09 | -0.10 | 0.56 | 0.61 |
| Victim intoxication | -0.48 | -0.49 | 0.02 | 0.01 | -0.37 | -0.36 | 0.16 | 0.16 |
| Victimization history ^b^ | 0.26 | 0.26 | 0.75 | 0.75 | 0.19 | 0.19 | 0.71 | 0.72 |
| *SRQ Unsupportive Acknowledgement* |  |  |  |  |  |  |  |  |
| Assaulted by someone close^a^ | -0.09 | -0.1 | 0.6 | 0.62 | -0.17 | -0.19 | 0.53 | 0.54 |
| Unwanted penetration | -0.55 | -0.55 | 0.11 | 0.09 | -0.62 | -0.62 | 0.05 | 0.06 |
| Physical force/threats to harm | -0.01 | -0.02 | 0.51 | 0.52 | -0.17 | -0.15 | 0.44 | 0.46 |
| Physical violence | 0.06 | 0.04 | 0.65 | 0.67 | 0.00 | 0.01 | 0.69 | 0.69 |
| Victim intoxication | -0.40 | -0.39 | 0.12 | 0.12 | -0.26 | -0.26 | 0.31 | 0.31 |
| Victimization history ^b^ | 0.05 | 0.06 | 0.56 | 0.57 | 0.003 | 0.01 | 0.55 | 0.59 |
| *SRQ Positive reactions* |  |  |  |  |  |  |  |  |
| Assaulted by someone close^a^ | -0.84 | -0.81 | -0.10 | -0.12 | -0.76 | -0.74 | 0.002 | -0.04 |
| Unwanted penetration | -0.31 | -0.29 | 0.42 | 0.41 | -0.28 | -0.29 | 0.45 | 0.47 |
| Physical force/threats to harm | -0.43 | -0.42 | 0.15 | 0.14 | -0.44 | -0.44 | 0.22 | 0.20 |
| Physical violence | -0.32 | -0.3 | 0.34 | 0.32 | -0.26 | -0.21 | 0.49 | 0.43 |
| Victim intoxication | -0.21 | -0.22 | 0.36 | 0.36 | -0.29 | -0.29 | 0.33 | 0.34 |
| Victimization history ^b^ | -0.72 | -0.71 | -0.17 | -0.16 | -0.65 | -0.64 | -0.05 | -0.06 |
| *Posttraumatic Stress symptoms* |  |  |  |  |  |  |  |  |
| Age | 0.01 | 0.01 | 0.04 | 0.04 | 0.00 | 0.00 | 0.03 | 0.03 |
| National background | -0.33 | -0.31 | 0.49 | 0.45 | -0.35 | -0.32 | 0.39 | 0.36 |
| Help-seeking | 0.08 | 0.08 | 0.63 | 0.63 | 0.07 | 0.06 | 0.62 | 0.62 |
| Time since assault (ref 3-6 months): |  |  |  |  |  |  |  |  |
| 1-6 days | -0.58 | -0.58 | 0.32 | 0.31 | -0.49 | -0.47 | 0.40 | 0.4 |
| 1-2 weeks | -0.59 | -0.50 | 0.34 | 0.26 | -0.67 | -0.68 | 0.21 | 0.22 |
| 3-4 weeks | -0.61 | -0.63 | 0.37 | 0.36 | -0.58 | -0.68 | 0.32 | 0.22 |
| 1-3 months | -0.42 | -0.43 | 0.29 | 0.28 | -0.23 | -0.25 | 0.42 | 0.44 |
| Assaulted by someone close^a^ | -0.30 | -0.30 | 0.43 | 0.42 | -0.53 | -0.52 | 0.20 | 0.2 |
| Unwanted penetration | -0.29 | -0.29 | 0.41 | 0.42 | -0.37 | -0.35 | 0.31 | 0.32 |
| Physical force/threats to harm | 0.04 | 0.05 | 0.60 | 0.59 | -0.26 | -0.26 | 0.35 | 0.34 |
| Physical violence | 0.17 | 0.15 | 0.79 | 0.76 | -0.15 | -0.21 | 0.53 | 0.54 |
| Victim intoxication | -0.52 | -0.52 | 0.03 | 0.02 | -0.32 | -0.31 | 0.24 | 0.21 |
| Victimization history | 0.24 | 0.24 | 0.78 | 0.77 | -0.01 | -0.01 | 0.57 | 0.55 |
| SRQ Turning against | 0.17 | 0.12 | 0.48 | 0.47 | -0.28 | -0.36 | 0.19 | 0.24 |
| SRQ Unsupportive acknowledgement | 0.26 | 0.28 | 0.56 | 0.54 | 0.14 | 0.13 | 0.57 | 0.58 |
| SRQ Positive reactions | -0.17 | -0.17 | 0.12 | 0.11 | -0.13 | -0.13 | 0.19 | 0.18 |
| Social support | -0.37 | -0.35 | -0.11 | -0.12 | -0.28 | -0.27 | 0.01 | 0.00 |
| *Anxiety/depression symptoms* |  |  |  |  |  |  |  |  |
| Age | 0.00 | 0.00 | 0.03 | 0.03 | 0.00 | 0.00 | 0.02 | 0.02 |
| National background | -0.21 | -0.19 | 0.43 | 0.38 | -0.26 | -0.26 | 0.35 | 0.34 |
| Help-seeking | -0.01 | -0.01 | 0.42 | 0.43 | -0.12 | -0.14 | 0.33 | 0.33 |
| Time since assault (ref 3-6 months): |  |  |  |  |  |  |  |  |
| 1-6 days | -0.04 | 0.01 | 0.66 | 0.60 | 0.01 | 0.04 | 0.75 | 0.72 |
| 1-2 weeks | -0.25 | -0.21 | 0.48 | 0.43 | -0.31 | -0.31 | 0.41 | 0.40 |
| 3-4 weeks | -0.41 | -0.52 | 0.36 | 0.39 | -0.32 | -0.38 | 0.42 | 0.44 |
| 1-3 months | -0.31 | -0.31 | 0.25 | 0.26 | -0.23 | -0.25 | 0.31 | 0.33 |
| Assaulted by someone close^a^ | -0.25 | -0.30 | 0.33 | 0.34 | -0.29 | -0.35 | 0.31 | 0.33 |
| Unwanted penetration | -0.35 | -0.34 | 0.21 | 0.22 | -0.38 | -0.39 | 0.18 | 0.19 |
| Physical force/threats to harm | -0.12 | -0.11 | 0.32 | 0.31 | -0.29 | -0.26 | 0.21 | 0.20 |
| Physical violence | -0.17 | -0.16 | 0.33 | 0.30 | -0.34 | -0.33 | 0.22 | 0.20 |
| Victim intoxication | -0.35 | -0.35 | 0.08 | 0.07 | -0.33 | -0.33 | 0.13 | 0.13 |
| Victimization history | 0.13 | 0.14 | 0.56 | 0.56 | -0.10 | -0.12 | 0.38 | 0.39 |
| SRQ Turning against | 0.07 | 0.09 | 0.32 | 0.31 | -0.09 | -0.09 | 0.29 | 0.32 |
| SRQ Unsupportive acknowledgement | 0.05 | 0.05 | 0.30 | 0.29 | -0.12 | -0.12 | 0.23 | 0.23 |
| SRQ Positive reactions | -0.06 | -0.06 | 0.17 | 0.17 | 0.03 | 0.02 | 0.29 | 0.28 |
| Social support | -0.31 | -0.30 | -0.11 | -0.11 | -0.32 | -0.32 | -0.08 | -0.08 |

Results shown for unadjusted and fully adjusted models. Results not shown for Model 1 (PTSD and anxiety/depression symptoms).
